# Evidence for factors associated with diet and physical activity in African and Caribbean countries

**DOI:** 10.2471/BLT.20.269308

**Published:** 2021-04-01

**Authors:** Eleanor Turner-Moss, Ahmed Razavi, Nigel Unwin, Louise Foley, on behalf of the Global Diet and Activity Research Group and Network

**Affiliations:** a MRC Epidemiology Unit, University of Cambridge School of Clinical Medicine, Box 285, Institute of Metabolic Science, Cambridge Biomedical Campus, Cambridge, CB2 0QQ, England.; b Global Public Health Division, Public Health England, London, England.

## Abstract

**Objective:**

To identify and describe summarized evidence on factors associated with diet and physical activity in low- and middle-income countries in Africa and the Caribbean by performing a scoping review of reviews.

**Methods:**

We searched the Medline®, LILACS, Scopus, Global Health and Web of Science databases for reviews of factors associated with diet or physical activity published between 1998 and 2019. At least 25% of studies in reviews had to come from African or Caribbean countries. Factors were categorized using Dahlgren and Whitehead’s social model of health. There was no quality appraisal.

**Findings:**

We identified 25 reviews: 13 on diet, four on physical activity and eight on both. Eighteen articles were quantitative systematic reviews. In 12 reviews, 25–50% of studies were from Africa or the Caribbean. Only three included evidence from the Caribbean. Together, the 25 reviews included primary evidence published between 1926 and 2018. Little of the summarized evidence concerned associations between international health or political factors and diet or associations between any factor and physical activity across all categories of the social model of health.

**Conclusion:**

The scoping review found a wide range of factors reported to be associated with diet and physical activity in Africa and the Caribbean, but summarized evidence that could help inform policies encouraging behaviours linked to healthy diets and physical activity in these regions were lacking. Further reviews are needed to inform policy where the evidence exists, and to establish whether additional primary research is needed.

## Résumé

**Objectif:**

Identifier et décrire les synthèses de données probantes consacrées aux facteurs liés au régime alimentaire et à l'activité physique dans les pays à faible et moyen revenu en Afrique et dans les Caraïbes, en examinant la portée des revues.

**Méthodes:**

Nous avons examiné les bases de données Medline®, LILACS, Scopus, Global Health et Web of Science en quête de revues publiées entre 1998 et 2019, consacrées aux facteurs liés au régime ou à l'activité physique. Au moins 25% des études citées dans ces revues devaient provenir d'Afrique ou des Caraïbes. Nous avons ensuite classé les facteurs à l'aide du modèle social de santé Dahlgren et Whitehead. Aucune évaluation de la qualité n'a été effectuée.

**Résultats:**

Nous avons repéré 25 revues: 13 sur le régime, quatre sur l'activité physique et huit réunissant les deux thèmes. Dix-huit articles étaient des revues systématiques quantitatives. Dans 12 revues, 25 à 50% des études avaient été réalisées en Afrique ou dans les Caraïbes. Seulement trois contenaient des éléments de preuve relatifs aux Caraïbes. Prises dans leur ensemble, les 25 revues renfermaient des preuves primaires publiées entre 1926 et 2018. Rares étaient les synthèses de données probantes consacrées aux liens entre la santé internationale ou les facteurs politiques d'une part, et le régime alimentaire de l'autre, ou encore entre n'importe quel facteur et l'activité physique dans toutes les catégories du modèle social de santé.

**Conclusion:**

L'examen de la portée a permis de découvrir un large éventail de facteurs considérés comme en lien avec le régime alimentaire et l'activité physique en Afrique et dans les Caraïbes. Toutefois, il manquait une synthèse des données probantes, qui aurait pu contribuer à orienter les politiques destinées à encourager les comportements favorisant un régime alimentaire sain et la pratique d'une activité physique dans ces régions. D'autres revues sont nécessaires pour fournir des informations aux politiques lorsqu'il existe une preuve, et pour déterminer si une recherche préliminaire supplémentaire est requise.

## Resumen

**Objetivo:**

Identificar y describir las pruebas resumidas sobre los factores asociados a la dieta y la actividad física en los países de ingresos bajos y medios de África y el Caribe mediante la realización de una revisión del alcance de las revisiones.

**Métodos:**

Realizamos búsquedas en las bases de datos Medline®, LILACS, Scopus, Global Health y Web of Science de revisiones de factores asociados a la dieta o la actividad física publicadas entre 1998 y 2019. Al menos el 25% de los estudios de las revisiones debían proceder de África o el Caribe. Los factores se clasificaron utilizando el modelo social de salud de Dahlgren y Whitehead. No hubo evaluación de la calidad.

**Resultados:**

Identificamos 25 revisiones: 13 sobre la dieta, cuatro sobre la actividad física y ocho sobre ambas. Dieciocho artículos eran revisiones sistemáticas cuantitativas. En 12 revisiones, entre el 25 y el 50% de los estudios eran de África o el Caribe. Solo tres incluyeron pruebas del Caribe. En conjunto, las 25 revisiones incluyeron evidencia primaria publicada entre 1926 y 2018. Pocas de las pruebas resumidas se referían a las asociaciones entre los factores políticos o de salud internacionales y la dieta o las asociaciones entre cualquier factor y la actividad física en todas las categorías del modelo social de salud.

**Conclusión:**

En la revisión del alcance encontramos una gran variedad de factores que, según los informes, se asocian con la dieta y la actividad física en África y el Caribe, pero carecemos de pruebas resumidas que puedan ayudar a informar las políticas que fomentan los comportamientos relacionados con las dietas saludables y la actividad física en estas regiones. Es necesario realizar más revisiones para informar a las políticas sobre los puntos en los que existen pruebas y sobre la necesidad de realizar investigaciones primarias adicionales.

## ملخص

الغرض تحديد ووصف الدليل الموجز على العوامل المرتبطة بالنظام الغذائي والنشاط البدني في البلدان منخفضة الدخل ومتوسطة الدخل في أفريقيا ومنطقة البحر الكاريبي، عن طريق إجراء مراجعة عن كثب للمراجعات.

الطريقة قمنا بالبحث في قواعد بيانات Medline®، وLILACS، وScopus، وGlobal Health، وWeb of Science، عن لمراجعات للعوامل المرتبطة بالنظام الغذائي أو النشاط البدني، والمنشورة بين عامي 1998 و2019. كان يجب أن تأتي 25% على الأقل من دراسات المراجعات من إفريقيا أو منطقة البحر الكاريبي. تم تصنيف العوامل باستخدام نموذج دالجرين ووايتهيد الاجتماعي للصحة، ولم يكن هناك تقييم للجودة.

النتائج لقد حددنا 25 مراجعة، 13 منها عن النظام الغذائي، وأربعة عن النشاط البدني، وثمانية منها عنهما معًا. كانت ثماني عشرة مقالة عبارة عن مراجعات منهجية كمية. في 12 مراجعة، كانت 25% إلى 50% من الدراسات من أفريقيا أو منطقة البحر الكاريبي. وشملت ثلاثة منها فقط دليلاً من منطقة البحر الكاريبي. تضمنت المراجعات الـ 25، جنباً إلى جنب، أدلة أولية تم نشره بين عامي 1926 و2018. القليل من الدليل الموجز تعلق بالارتباطات بين العوامل الصحية أو السياسية الدولية، والنظام الغذائي أو الارتباطات بين أي عامل والنشاط البدني عبر كل فئات النموذج الاجتماعي للصحة.

الاستنتاج وجدت المراجعة عن كثب مجموعة واسعة من العوامل التي تم الإبلاغ عن ارتباطها بالنظام الغذائي والنشاط البدني في إفريقيا ومنطقة البحر الكاريبي، إلا أن الأدلة الموجزة الذي يمكن أن يساعد في توجيه السياسات التي تشجع السلوكيات المرتبطة بالنظم الغذائية الصحية والنشاط البدني في هذه المناطق، كانت مفقودة. هناك حاجة إلى مزيد من المراجعات لتوجيه السياسة حينما يكون هناك دليل، وما إذا كانت هناك حاجة إلى بحث أولي إضافي.

## 摘要

**目的:**

旨在通过对综述文献进行概况性评价，确定和说明非洲和加勒比地区中低收入国家饮食和身体活动相关因素的汇总证据。

**方法:**

我们搜索了 Medline®、LILACS、Scopus、全球健康和科学网数据库，以查看 1998 年至 2019 年期间发布的饮食或身体活动相关因素综述文献。在这些综述文献中，至少有 25％ 的研究项目来自非洲或加勒比地区。使用 Dahlgren 和 Whitehead 的健康社会模型对因素进行了分类。但并未进行质量鉴定。

**结果:**

我们确定了 25 份综述文献：其中 13 份与饮食有关，4 份关于身体活动，8 份与两者均有关。十八篇文章采用了定量系统评价方法。在 12 份综述文献中，25-50% 的研究项目来自非洲或加勒比地区。其中仅三份证据来自加勒比地区。这 25 份综述文献整体涵括了 1926 年至 2018 年期间公布的主要证据。汇总证据很少涉及国际健康或政治因素与饮食之间的关联性，或健康社会模型所有类别中任何因素与身体活动之间的关联性。

**结论:**

通过概况性评价，我们发现非洲和加勒比地区众多因素均与饮食和身体活动有关，但缺少汇总证据来帮助推行适当政策，以鼓励在这些地区实施与健康饮食和身体活动有关的行为。需要进一步查询综述文献，以基于已有证据推行适当政策并确定是否需要开展其他初步研究工作。

## Резюме

**Цель:**

Выявить и описать обобщенные данные о факторах, связанных с питанием и физической активностью, в странах Африки и Карибского бассейна с низким и средним уровнем доходов путем составления обзора проведенных исследований.

**Методы:**

Авторы выполнили поиск информации в базах данных Medline®, LILACS, Scopus, Global Health и Web of Science, опубликованной в период с 1998 по 2019 год, с целью изучения факторов, связанных с питанием или физической активностью. Минимум 25% исследований в обзорах должны были проводиться в странах Африки или Карибского бассейна. Эти факторы были классифицированы с использованием социальной модели факторов здоровья Дальгрена и Уайтхед. Оценка качества не проводилась.

**Результаты:**

Было составлено 25 обзоров: 13 по питанию, 4 по физической активности и 8 по обоим факторам. Восемнадцать статей являлись количественными систематическими обзорами. В 12 обзорах от 25 до 50% исследований приходилось на страны Африки и Карибского бассейна. Только три из них содержали данные по странам Карибского бассейна. В общей сложности 25 обзоров содержали данные первичных исследований, опубликованные в период с 1926 по 2018 год. Малый процент обобщенных данных касался взаимосвязей между международным здравоохранением или политическими факторами и питанием либо взаимосвязей между любыми факторами и физической активностью по всем категориям социальной модели здоровья.

**Вывод:**

Обзор проведенных исследований выявил широкий спектр факторов, связанных с питанием и физической активностью, в странах Африки и Карибского бассейна, однако обобщенные данные, которые могли бы способствовать разработке политики поощрения здорового питания и физической активности, по этим регионам отсутствовали. Дальнейшие обзоры необходимы для подкрепления политических мер в случае наличия таких данных, а также для определения необходимости проведения дополнительных первичных исследований.

## Introduction

Almost three quarters of deaths from noncommunicable disease occur in low- and middle-income countries, particularly in Africa and the Caribbean.^1^ Moreover, the burden of noncommunicable disease in the World Health Organization’s (WHO) African Region is expected to exceed that of communicable disease by 2030.^2^ Premature death from noncommunicable disease in these regions is relatively common; for example, the probability of dying between the ages of 30 and 70 years from noncommunicable disease is 12% in the United Kingdom of Great Britain and Northern Ireland, whereas, in Kenya, Cameroon and South Africa, it is 18%, 20% and 27%, respectively, and in the Caribbean, it ranges from 17% in Jamaica to 37% in Guyana.^1^

Studies consistently show that an unhealthy diet and physical inactivity are the leading modifiable behavioural risk factors for the four primary noncommunicable diseases: type 2 diabetes, cardiovascular disease, cancer and chronic respiratory disease.^3^ Clear recommendations have been made by WHO for a healthy diet (i.e. high intake of fruit, vegetables and fibre and low intake of fat, sugar and salt) and physical activity (e.g. at least 150 minutes of moderate-intensity activity per week for adults).^4^^,^^5^ According to 2019 Global Burden of Disease data,^6^ the percentage of deaths from noncommunicable disease directly attributable to diet was 15.6% in Africa and 15.3% in the Caribbean; the percentage directly attributable to low physical activity was 2.2% in Africa and 3.7% in the Caribbean.

In both Africa and the Caribbean there are ongoing regional and national policy initiatives on noncommunicable disease, consistent with WHO’s *Global action plan for the prevention and control of noncommunicable diseases 2013–2020*.^4^ In Africa, these include the regional 2011 Brazzaville Declaration and national policy initiatives.^7^ In the Caribbean, the 2007 Port of Spain Declaration on noncommunicable diseases was a first for lower-middle-income regions. This declaration provided a framework for the development and implementation of policies on the prevention and control of noncommunicable disease, both regionally and nationally. An evaluation of the Port of Spain Declaration in 2018 found that taking effective measures to address the distal (or upstream) determinants of an unhealthy diet and physical inactivity (e.g. cultural and environmental conditions) remained challenging,^8^ although new initiatives, such as taxing sugar-sweetened beverages, were being implemented.

Behaviours associated with a healthy diet and physical activity are core contributors to good health and, thus, the ability to participate in these behaviours can be viewed as a universal right. These behaviours are shaped by a range of factors, including: (i) international policies and politics; (ii) socioeconomic, cultural and environmental conditions; (iii) living and working conditions; (iv) social and community networks; and (v) more proximal individual factors (e.g. age and sex).^4^^,^^5^^,^^9^ Evidence on factors associated with these behaviours, on their distribution across different population groups and on whether they are modifiable is important for understanding the drivers of disease burden, for predicting future trends and for identifying targets for interventions and policy changes.

Most existing research summaries on the determinants of diet and physical activity come from high-income countries. Consequently, the generalizability of their findings to Africa and the Caribbean is questionable and evidence is needed from low- and middle-income countries to inform research, interventions and policy development.^10^^,^^11^ Scoping reviews adopt a systematic approach to map published evidence on a topic, summarize the main themes and highlight knowledge gaps.^12^ We chose to conduct a scoping review of reviews because systematic reviews and meta-analyses provide the highest level of evidence on which to draw evidence-based conclusions.

The principle aim of our study was to identify and summarize existing reviews on a broad range of factors associated with diet and physical activity in low- and middle-income countries in Africa and the Caribbean. A secondary aim was to identify gaps in the current evidence. Our review was conducted as part of an initial scoping exercise for the Global Diet and Activity Research Network,^13^ which is a collaboration of researchers in the Caribbean, Cameroon, Kenya, South Africa and the United Kingdom. The overall goal of the network is to generate evidence on the determinants of diet and physical activity to inform noncommunicable disease prevention in Africa and the Caribbean.

## Methods

This scoping review of reviews was conducted according to a previously described method^12^ and followed reporting guidance in the preferred reporting items for systematic reviews and meta-analyses extension for scoping reviews.^14^ A review protocol was developed beforehand and was consistent with the scoping review method.^15^ The review question and the study selection criteria were developed iteratively as familiarity with the literature increased.

We searched the Medline®, LILACS, Scopus, Global Health and Web of Science databases for reviews of factors associated with physical activity and dietary behaviour in Africa and the Caribbean that were published between January 1998 and December 2019. A search was carried out in April 2018 and, again, in December 2020 to include literature to the end of 2019. No author was contacted to provide additional information and no grey literature was included because our aim was to identify peer-reviewed evidence syntheses.

Our full search strategy is detailed in Box 1 (available at: http://www.who.int/bulletin/volumes/99/6/20-269308). In brief, we combined search terms in sets: (i) diet (e.g. diet, nutrition, food intake, fruit, vegetables, fat, sugar, salt and junk food); (ii) physical activity (e.g. walking, manual labour and screen time); (iii) determinants (e.g. risk factors, correlations and demographic factors); (iv) low- and middle-income countries, with specific terms for African and Caribbean countries; and (v) reviews (i.e. reviews of quantitative or qualitative studies).

Box 1 **Search strategies, scoping review of reviews of factors associated with diet and physical activity in Africa and the Caribbean, 1998–2019**

**Medline® search strategy**

1. diet.mp. OR exp DIET

2. exp NUTRITION DISORDERS/ OR nutrition*.mp.

3. food intake.mp. OR exp Eating/

4. exp Feeding Behavior/ OR eating behavio?r*.mp.

5. junk* food*.mp.

6. (calori* adj2 intake*).mp.

7. meat consumption.mp.

8. (high adj2 (fat* OR salt* OR sugar*)).mp.

9. malnutrition.mp. OR exp MALNUTRITION/

10. exp Malnutrition/ OR malnourish*.mp.

11. (fruit AND veg*).mp.

12. exp Energy Intake/ OR energy intake*.mp.

13. (physical* adj2 activ*).mp.

14. exp Exercise/

15. exercis*.mp.

16. (active adj2 (living OR transport* OR travel*)).mp.

17. walk*.mp. OR exp Walking/

18. (bike OR bicycl* OR biking).mp.

19. exp SEDENTARY LIFESTYLE/ OR exp Physical Exertion/ OR sedentary.mp.

20. (physical* adj2 exert*).mp.

21. (screen time OR screentime).mp.

22. manual labo?r*.mp.

23. subsistence.mp.

24. mobil*.mp.

25. or/1–24

26. determinant*.mp. OR exp “SOCIAL DETERMINANTS OF HEALTH”/

27. exp SOCIOECONOMIC FACTORS/ OR socioeconomic*.mp.

28. associat*.mp.

29. correlat*.mp.

30. (policy OR policies).mp.

31. legislat*.mp.

32. exp Risk Factors/ OR risk factor*.mp.

33. built environment.mp. OR exp Environment Design/

34. exp SOCIAL ENVIRONMENT/ OR exp ENVIRONMENT/ OR environment*.mp.

35. cultur*.mp. OR exp Culture/

36. ethnograph*.mp.

37. psychosocial*.mp.

38. exp Demography/ OR demograph*.mp. OR exp Population Dynamics/ OR exp Population Characteristics/

39. exp Epidemiology/ OR exp Epidemiologic Studies/ OR exp Epidemiologic Methods/ OR exp Epidemiological Monitoring/ OR epidemiolog*.mp.

40. (cohort* OR longitudinal* OR observation*).mp.

41. or/26–40

42. Developing Countries.sh,kf.

43. ((developing OR less* developed OR under developed OR underdeveloped OR middle income OR low* income OR underserved OR under served OR deprived OR poor*) adj (countr* OR nation? OR population? OR world)).ti,ab.

44. ((developing OR less* developed OR under developed OR underdeveloped OR middle income OR low* income) adj (economy OR economies)).ti,ab.

45. (low* adj (gdp OR gnp OR gross domestic OR gross national)).ti,ab.

46. (low adj3 middle adj3 countr*).ti,ab.

47. (lmic OR lmics OR third world OR lami countr*).ti,ab.

48. transitional countr*.ti,ab.

49. review.pt.

50. review*.ab,ti.

51. 49 OR: 50

52. (Africa OR Caribbean OR West Indies).hw,ti,ab,cp.

53. exp AFRICA/

54. exp Caribbean Region/

55. (Africa OR Caribbean OR Sub-Sahara* OR “Sub Sahara*” OR Algeria OR Angola OR Belize OR Benin OR Botswana OR “Burkina Faso” OR Burundi OR “Cabo Verde” OR “Cape verde” OR Cameroon OR “Central African Republic” OR Chad OR Comoros OR Comores OR Comoro OR Congo OR “Cote d'Ivoire” OR Cuba OR Djibouti OR Dominica OR “Dominican Republic” OR Egypt OR Eritrea OR Ethiopia OR Gabon OR Gambia OR Ghana OR Grenada OR Grenadines OR Guinea OR “Guinea Bisau” OR Guyana OR Haiti OR Jamaica OR Kenya OR Lesotho OR Liberia OR Libya OR Madagascar OR Malawi OR Mali OR Mauritania OR Mauritius OR Morocco OR Mozambique OR Namibia OR Niger OR Nigeria OR Principe OR Rwanda OR Ruanda OR “Sao Tome” OR Senegal OR “Sierra Leone” OR Somalia OR “South Africa” OR “South Sudan” OR “St Lucia” OR “St Vincent” OR Sudan OR Surinam OR Suriname OR Swaziland OR Tanzania OR Togo OR Tunisia OR Uganda OR Zambia OR Zimbabwe).tw.

56. or/52–55

57. (non-infectious* OR noncommunicable* OR NCD OR non-communicable*).mp.

58. 42 OR: 43 OR: 44 OR: 45 OR: 46 OR: 47 OR: 48 OR: 56

59. 25 OR: 57

60. 59 AND 41 AND 58 AND 51

61. review.m_titl.

62. 59 AND 41 AND 58 AND 61

63. ('scoping review' OR 'systematic review' OR 'narrative review' OR 'literature review' OR 'evidence review' OR 'mixed methods review' OR 'realist review' OR 'realist synthesis' OR 'meta-ethnography' OR 'meta ethnography').ab,ti.

64. 59 AND 41 AND 58 AND 63

**Search strategy for other databases**

#1 TS = (diet)

#2 TS = nutrition*

#3 TS = food intake

#4 TS = eating behavio?r*

#5 TS = (junk* food*)

#6 TS = (“calori* intake*”)

#7 TS = (meat consumption)

#8 TS = (“high fat*” OR “high salt*” OR “high sugar*”

#9 TS = malnutrition

#10 TS = malnourish*

#11 TS = (fruit* AND veg*)

#12 TS = (energy intake*)

#13 TS = (“physical* activ*”)

#14 TS = exercis*

#15 TS = (“active living” OR “active transport*” OR “active travel*”)

#16 TS = walk*

#17 TS = (bike OR bicycl* OR biking)

#18 TS = sedentary lifestyle

#19 TS = physical* exert*

#20 TS = (screentime OR “screen time”)

#21 TS = manual labo?r

#22 TS = subsistence

#23 TS = mobilisa*

#24 #23 OR #22 OR #21 OR #20 OR #19 OR #18 OR #17 OR #16 OR #15 OR #14 OR #13 OR #12 OR #11 OR #10 OR #9 OR #8 OR #7 OR #6 OR #5 OR #4 OR #3 OR #2 OR #1

#25 TS = (determinant* OR socioeconomic* OR associat* OR correlat* OR policy OR policies OR legislat* OR risk factor* OR built environment OR environment* OR cultur* OR ethnograph* OR psychosocial* OR demograph* OR epidemiolog* OR cohort* OR longitudinal* OR observation*)

#26 TS = (“scoping review” OR “systematic review” OR “narrative review” OR “literature review” OR “evidence review” OR “mixed methods review” OR “realist review” OR “realist synthesis” OR “meta-ethnography” OR “meta ethnography”)

#27 TS = (Africa OR Caribbean OR “West Indies”)

#28 TS = (Africa OR Caribbean OR Sub-Sahara* OR “Sub Sahara*” OR Algeria OR Angola OR Belize OR Benin OR Botswana OR “Burkina Faso” OR Burundi OR “Cabo Verde” OR “Cape verde” OR Cameroon OR “Central African Republic” OR Chad OR Comoros OR Comores OR Comoro OR Congo OR “Cote d'Ivoire” OR Cuba OR Djibouti OR Dominica OR “Dominican Republic” OR Egypt OR Eritrea OR Ethiopia OR Gabon OR Gambia OR Ghana OR Grenada OR Grenadines OR Guinea OR “Guinea Bisau” OR Guyana OR Haiti OR Jamaica OR Kenya OR Lesotho OR Liberia OR Libya OR Madagascar OR Malawi OR Mali OR Mauritania OR Mauritius OR Morocco OR Mozambique OR Namibia OR Niger OR Nigeria OR Principe OR Rwanda OR Ruanda OR “Sao Tome” OR Senegal OR “Sierra Leone” OR Somalia OR “South Africa” OR “South Sudan” OR “St Lucia” OR “St Vincent” OR Sudan OR Surinam OR Suriname OR Swaziland OR Tanzania OR Togo OR Tunisia OR Uganda OR Zambia OR Zimbabwe)

#29 TS = “developing countries”

#30 TS = (“developing countr*” OR “less* developed countr*” OR “under developed countr*” OR “underdeveloped countr*” OR “middle income countr*” OR “low* income countr*” OR “underserved countr*” OR “under served countr*” OR “deprived countr*” OR “poor countr*” OR “developing nation**” OR “less* developed nation*” OR “under developed nation*” OR “underdeveloped nation*” OR “middle income nation*” OR “low* income nation*” OR “underserved nation*” OR “under served nation*” OR “deprived nation*” OR “poor nation*” OR “developing population*” OR “less* developed population*” OR “under developed population*” OR “underdeveloped population*” OR “middle income population*” OR “low* income population*” OR “underserved population*” OR “under served population*” OR “deprived population*” OR “poor population*” OR “developing world*” OR “less* developed world*” OR “under developed world*” OR “underdeveloped world*” OR “middle income world*” OR “low* income world*” OR “underserved world*” OR “under served world*” OR “deprived world*” OR “poor world*”)

#31 TS = (low* gdp OR low* GNP OR low* gross domestic OR low* gross national)

#32 TS = low middle countr*

#33 TS = (lmic OR lmics OR third world OR lami countr*)

#34 TS = transitional countr*

#35 #34 OR #33 OR #32 OR #31 OR #30 OR #29 OR #28 OR #27

#36 #35 AND #26 AND #25 AND #24

Note: Each numbered line was run as a separate search. Then, the searches were combined in different ways using Boolean operators and the line numbers for each search.

Reviews were eligible for inclusion if they provided summaries of primary research on factors associated with diet and physical activity and at least 25% of studies included were conducted in low- or middle-income African or Caribbean countries. Reviews could include quantitative or qualitative evidence from observational or interventional studies. We excluded literature reviews that: (i) explored how diet or physical activity shaped health outcomes or disease burden; (ii) reported only health outcomes; (iii) dealt primarily with health-system care models; (iv) focused on migrant groups or ethnic minorities in high-income countries; (v) related to humanitarian crises or natural disasters; (vi) considered only nutritional biomarkers, without an accompanying assessment of diet; (vii) addressed breastfeeding as a determinant of diet in infants; or (viii) were not published in English.

Citations identified by the search strategy were first imported into Rayyan QCRI systematic review software (Qatar Computing Research Institute, Doha, Qatar) and any duplicates were removed. Working in two pairs, we double-screened the titles and abstracts of all citations. If there was a conflict, all authors conferred, with two authors acting as arbiters. Then, the full texts of selected reviews were retrieved and, again working in two pairs, we double-screened all texts. Any disagreement was discussed with reference to the eligibility criteria, with any one of three authors acting as arbiter.

We examined the final set of selected reviews to determine which data items should be abstracted and their format. The abstraction fields identified included: (i) the citation; (ii) the type of review; (iii) the number and type of studies in the review, including specifically the number and type of studies conducted in Africa and the Caribbean; (iv) the review setting (e.g. urban or rural); (v) the target population group (e.g. children or adults); (vi) factors associated with diet or physical activity; (vii) outcomes; and (viii) the main findings. Three authors entered data into a pretested data abstraction form. Where possible, data and conclusions specifically relevant to African and Caribbean countries were abstracted separately. We used Dahlgren and Whitehead’s social model of health to categorize and conceptualize both distal factors (e.g. international policies and politics, and socioeconomic, cultural and environmental conditions) and proximal factors (e.g. living and working conditions, social and community networks and individual factors such as age and sex).^9^ The abstracted data were summarized and tabulated.

Our scoping review of reviews describes the results, discussions and conclusions of the selected reviews, not of the primary studies underlying them. Moreover, as is common in scoping reviews, there was no appraisal of the quality of the reviews. Hypothesized or putative explanations for relationships identified in the reviews were included in our summary only if supported by a synthesis of the underlying primary studies.

## Results

**Fig. 1:**
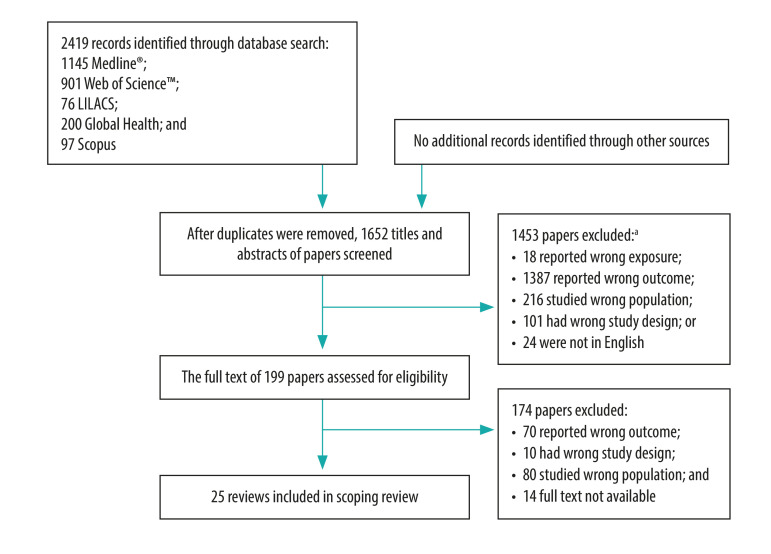
Selection of reviews for the scoping review of factors associated with diet and physical activity in Africa and the Caribbean, 1998–2019

^a^ Records could be excluded for several reasons.

**Fig. 2:**
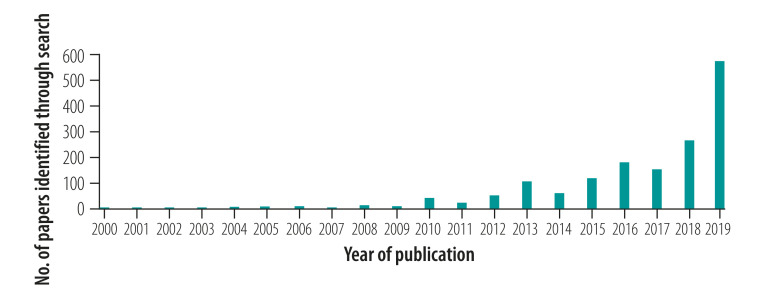
No. of papers identified in search for reviews of factors associated with diet and physical activity in Africa and the Caribbean, by year of publication, 1998–2019

Note: Duplicates were removed.

The database searches identified 1652 unique citations whose titles and abstracts were screened (Fig. 1). Of these, 199 were selected for full text screening and, finally, 25 reviews were included in our scoping review.^16^^–^^40^ The number of papers identified increased markedly over time (Fig. 2), with nine of the 25 selected reviews being published first in 2019.^25^^–^^28^^,^^36^^–^^40^ The detailed characteristics of the 25 reviews are listed in Table 1 (available at: http://www.who.int/bulletin/volumes/99/6/20-269308).

**Table 1:** Reviews included in scoping review of factors associated with diet and physical activity in Africa and the Caribbean, 1998–2019

Review author (publication year)	Publication years of studies in review	Type of review	No. studies in review	No. studies in Africa or the Caribbean	Review setting	African or Caribbean countries in studies reviewed	Population group studied	Factors associated with diet or physical activity	Notes on factors	Outcomes	Notes on outcomes
**≥ 50% of studies in review from Africa or the Caribbean**											
Abubakari and Bhopal (2008)^16^	1964–2003	Systematic review of quantitative studies	16	16	Ghana and Nigeria	Ghana and Nigeria	Adults	Sex, socioeconomic status and urban or rural residence	NA	Physical activity	Additional outcomes of interest were diabetes prevalence and body composition
Raschke et al. (2008)^17^	1963–1969	Systematic review of historic data (design of included studies unclear)	6	6	East Africa	Kenya, Uganda and United Republic of Tanzania	Children and adults	Colonialization, natural environment and urbanization	(i) Cash-crop farming and replacement of indigenous crops; (ii) global food systems; (iii) urbanization; and (iv) destruction of natural ecosystems	Diet	(i) Food shortages; (ii) dependence on introduced or donated cereals; and (iii) loss of dietary diversity
Abubakari et al. (2009)^18^	1964–2003	Systematic review and meta-analysis of quantitative studies	15	15	West Africa	Burkina Faso, Cameroon, Côte d'Ivoire, Gambia, Ghana, Mali, Nigeria and Senegal	Adults	Age, sex and urban or rural residence	NA	Physical activity	An additional outcome of interest was diabetes prevalence
Larouche et al. (2014)^19^	1982–2013	Systematic review of quantitative studies	20^a^	20	Africa	Algeria, Botswana, Djibouti, Egypt, Ghana, Kenya, Libya, Malawi, Mauritius, Morocco, Namibia, Nigeria, Senegal, Seychelles, South Africa, Uganda, United Republic of Tanzania, Zambia and Zimbabwe	Children and young people	Socioeconomic status and urban or rural residence	NA	Physical activity	Active travel (walking, running or cycling for transport)
Muthuri et al. (2014)^20^	1967–2013	Systematic review of quantitative studies	71	71	Sub-Saharan Africa	Botswana, Cameroon, Côte d’Ivoire, Eswatini, Ethiopia, Ghana, Kenya, Mozambique, Namibia, Nigeria, Senegal, Seychelles, South Africa, Uganda, United Republic of Tanzania, Zambia and Zimbabwe	Children and young people	Age, socioeconomic status, sex and urban or rural residence	NA	Physical activity	Additional outcomes of interest were sedentary behaviour and physical fitness
Sobers-Grannum et al. (2015)^21^	2007–2013	Systematic review and meta-analysis of quantitative studies	50	50	Caribbean	Bahamas, Barbados, Cuba, Grenada, Guadeloupe, Jamaica, Puerto Rico, Saba, Suriname, Trinidad and Tobago and Virgin Islands (USA)	Children and adults	Ethnicity, socioeconomic status and sex	(i) Only findings on sex were summarized in the review; and (ii) socioeconomic status was derived from educational level, occupation and income	Diet and physical activity	(i) More studies were on physical activity than on diet; and (ii) additional outcomes of interest were body composition, tobacco smoking, metabolic syndrome and diabetes
Lew-Levy et al. (2017)^22^	1939–2015	Meta-ethnographic review of quantitative and qualitative studies	58	31	Hunter–gatherer societies	Botswana, Cameroon, Central African Republic, Democratic Republic of the Congo, Ethiopia, Madagascar, South Africa and United Republic of Tanzania	Children	Age, interventions, sex and social environment	Interventions included teaching, imitation and participation	Diet and physical activity	Self-sufficiency and subsistence skills for hunter–gatherer societies
Misselhorn and Hendriks (2017)^23^	1997–2014	Systematic review (design of included studies unclear)	169	169	South Africa (mainly rural areas)	South Africa	Assumed children and adults (unclear from article and appendices)	Access to food, food prices, urban or rural residence, socioeconomic status and sex	(i) Food stability (variability over time in supply and access); (ii) access to food (mediating factors of affordability, allocation and power relations); (iii) food utilization (nutritional value in terms of dietary quality, diversity and quantity, social value, food preparation and safety); and (iv) food availability (production, distribution and exchange)	Diet	Food insecurity
Pullar et al. (2018)^24^	1999–2015	Systematic review of quantitative intervention studies	29	15	Low- and middle-income countries	Burkina Faso, Burundi, Democratic Republic of the Congo, Egypt, Ethiopia, Ghana, Kenya, Malawi, Mali, Mozambique, Niger, Nigeria, Rwanda, Senegal, Uganda and United Republic of Tanzania	Children and adults	Interventions	(i) Poverty reduction; and (ii) development interventions targeting economic development, social inequalities, community engagement, agriculture, fisheries, water or sanitization, or human rights	Diet and physical activity	More studies were on diet than on physical activity
Abdurahman et al. (2019)^25^	2013–2018	Systematic review of quantitative studies	26	26	Ethiopia	Ethiopia	Infants and young children	Antenatal care, age, household composition, interventions, parental socioeconomic status, region and urban or rural residence	NA	Diet	Infant and young child feeding practices
Adom et al. (2019)^26^	2000–2018	Systematic review of quantitative intervention studies	10	10	Africa	South Africa and Tunisia	School children	Interventions	School-based interventions targeting diet, physical activity or weight	Diet and physical activity	An additional outcome of interest was weight
Gyawali et al. (2019)^27^	2003–2015	Systematic review of quantitative intervention studies	10	5	Low- and middle-income countries	Cameroon, South Africa and Uganda	Adults	Interventions	Community-based interventions for the prevention of type 2 diabetes	Diet and physical activity	Additional outcomes of interest were glycated haemoglobin levels, fasting blood glucose levels, blood pressure and weight
Klingberg et al. (2019)^28^	2009–2016	Systematic review of quantitative intervention studies	17	17	Africa	South Africa, Tunisia and Uganda	Children	Interventions	Primarily school or after school programmes	Diet and physical activity	(i) More studies were on physical activity than on diet; and (ii) additional outcomes of interest were anthropometry, physical fitness and screen time
**25–50% of studies in review from Africa or the Caribbean**											
Kristjansson et al. (2007)^29^	1926–2004	Systematic review and meta-analysis of quantitative intervention studies	18	5	Worldwide	Jamaica and Kenya	School children (low socioeconomic status)	Age, interventions, sex and socioeconomic status	Interventions were school meal programmes	Diet	Additional outcomes of interest were physical health, psychological health, behavioural variables and adverse events
Lee et al. (2013)^30^	1989–2010	Systematic review of quantitative studies	62	16	Low- and middle-income countries	Burkina Faso, Egypt, Ethiopia, Ghana, Jamaica, Kenya, Malawi, Morocco, Seychelles and South Africa	Pregnant women	Region and country	NA	Diet	NA
Johnston et al. (2015)^31^	1978–2014	Systematic review of quantitative and qualitative studies	89	27	Low- and middle-income countries (rural areas)	Unspecified countries in sub-Saharan Africa, the Middle East, North Africa and Latin America	Children and adults	Age, household composition, interventions, sex, social environment and socioeconomic status	Agricultural interventions and practices	Diet	(i) Diet and nutritional outcomes; and (ii) time use related to agriculture
Osendarp et al. (2016)^32^	2001–2014	Systematic review of quantitative studies	23	10	Low- and middle-income countries	Cambodia, Ethiopia, Malawi, South Africa, United Republic of Tanzania and Zimbabwe	Infants and young children	Age, interventions	The hypothetical optimization of intake of locally available foods	Diet	NA
Allen et al. (2017)^33^	1994–2015	Systematic review of quantitative studies	75	35	Low- and lower-middle-income countries	Benin, Burkina Faso, Chad, Comoros, Côte d'Ivoire, Democratic Republic of the Congo, Egypt, Eritrea, Eswatini, Ethiopia, Ghana, Kenya, Malawi, Mali, Mauritania, Morocco, Nigeria, Senegal, Togo, United Republic of Tanzania, Zambia and Zimbabwe	Children and adults	Age, sex and socioeconomic status	Socioeconomic status based on household or individual measures of income, wealth, assets, education, caste and occupation	Diet and physical activity	(i) More studies were on physical activity than on diet; and (ii) additional outcomes of interest were harmful use of alcohol and tobacco use
Graziose et al. (2018)^34^	2006–2016	Systematic review of quantitative intervention studies	18	5	Low- and middle-income countries	Burkina Faso, Kenya, Madagascar and Nigeria	Infants and young children	Interventions	Mass media and nutrition education interventions	Diet	Infants’ and young children’s feeding practices and related psychosocial factors, including the knowledge, attitudes and beliefs of caregivers
Kavle et al. (2018)^35^	2004–2015	Systematic review of quantitative and qualitative studies	23	8	Low- and middle-income countries	Burkina Faso, Egypt, Ethiopia, Kenya, Nigeria and Senegal	Pregnant and lactating women	Access to food, food prices, socioeconomic status and social environment	Specific barriers and facilitating factors associated with maternal diet during pregnancy and the postpartum period	Diet	NA
Abrahale et al. (2019)^36^	1985–2017	Systematic review	441	162	Worldwide	Unspecified countries in Africa	Children and adults	Street food availability and consumption	NA	Diet	An additional outcome of interest was food safety
Audate et al. (2019)^37^	1996–2017	Systematic review of quantitative and qualitative intervention studies	101	36	Worldwide	Benin, Botswana, Cameroon, Côte d'Ivoire, Eswatini, Ghana, Kenya, Lesotho, Malawi, Mozambique, Namibia, Nigeria, South Africa, Uganda, United Republic of Tanzania, Zambia and Zimbabwe	Children and adults (in urban areas)	Interventions	Urban agriculture	Diet	Additional outcomes of interest were food security, nutrition, social capital, health, sanitation, socioeconomic status, natural or physical environment, cultural connections and lifestyle
Boneya et al. (2019)^38^	2009–2017	Systematic review and meta-analysis of quantitative studies	17	6	Worldwide	Ethiopia, Senegal and Uganda	HIV-infected adults receiving antiretroviral therapy	Sex	NA	Diet	Food insecurity
Leandro et al. (2019)^39^	2001–2016	Systematic review of quantitative studies	11	4	Lower-middle- income countries	Ghana, Lesotho, Nigeria, Sudan and Uganda	Adolescents	Barriers to and enablers of obesogenic behaviour	NA	Diet and physical activity	Additional outcomes of interest were overweight and obesity
Webb Girard et al. (2020)^b,^^40^	2000–2017	Systematic review of quantitative intervention studies	64	23	Low- and middle-income countries	Egypt and unspecified countries in sub-Saharan Africa	Infants and young children	Interventions	Interventions to shift complementary feeding behaviours	Diet	Infant and young child feeding practices

HIV: human immunodeficiency virus; NA: not applicable.

^a^ An additional 19 studies assessed the psychometric properties of assessment tools. These were not restricted to Africa and predominantly included high-income countries.

^b^ First published online in 2019.

Thirteen reviews considered diet,^17^^,^^23^^,^^25^^,^^29^^–^^32^^,^^34^^–^^38^^,^^40^ four considered physical activity,^16^^,^^18^^–^^20^ and eight considered both.^21^^,^^22^^,^^24^^,^^26^^–^^28^^,^^33^^,^^39^ Eighteen reviews summarized quantitative evidence only,^16^^,^^18^^–^^21^^,^^24^^–^^30^^,^^32^^–^^34^^,^^38^^–^^40^ including four that conducted meta-analyses,^18^^,^^21^^,^^29^^,^^38^ and one that used modelling techniques.^32^ Four reviews incorporated both quantitative and qualitative evidence,^22^^,^^31^^,^^35^^,^^37^ including one that used a meta-ethnographic approach.^22^ The remaining three reviews presented a narrative summary of data and the design of the studies included was unclear.^17^^,^^23^^,^^36^

In 13 reviews,^16^^–^^28^ at least 50% of studies included were conducted in Africa or the Caribbean: nine focused on specific African regions or countries,^16^^–^^20^^,^^23^^,^^25^^,^^26^^,^^28^ whereas only one focused on the Caribbean.^21^ In the remaining 12 reviews,^29^^–^^40^ only 25–50% of studies came from Africa or the Caribbean – they tended to focus on low- and middle-income countries. A small number of countries were over-represented in the primary evidence: Kenya,^17^^,^^19^^,^^20^^,^^24^^,^^29^^,^^30^^,^^33^^–^^35^^,^^37^ Nigeria^16^^,^^18^^–^^20^^,^^24^^,^^33^^–^^35^^,^^37^^,^^39^ and South Africa,^19^^,^^20^^,^^22^^,^^23^^,^^26^^–^^28^^,^^30^^,^^32^^,^^37^ featured in 10 reviews each and Jamaica featured in all three reviews that included Caribbean countries.^21^^,^^29^^,^^30^

Of the 25 reviews, eight summarized evidence from both children and adults,^17^^,^^21^^,^^23^^,^^24^^,^^31^^,^^33^^,^^36^^,^^37^ six summarized evidence from adults only,^16^^,^^18^^,^^27^^,^^30^^,^^35^^,^^38^ and 11 summarized evidence from children only,^19^^,^^20^^,^^22^^,^^25^^,^^26^^,^^28^^,^^29^^,^^32^^,^^34^^,^^39^^,^^40^ including four related to infants.^25^^,^^32^^,^^34^^,^^40^ In addition, several reviews focused on specific settings or population groups, such as rural settings,^23^^,^^31^ socioeconomically disadvantaged areas,^29^ or pregnant or lactating women.^30^^,^^35^

Overall, the reviews summarized evidence from primary studies published between 1926 and 2018 – a 92-year time period. Fourteen reviews included only more recent studies (e.g. the past 20 years),^21^^,^^23^^–^^28^^,^^30^^,^^32^^,^^34^^,^^35^^,^^38^^–^^40^ whereas the other 11 either did not set a time period, or did not report time-limits, for the primary evidence.

### Outcomes

Dietary outcomes summarized in the reviews included: (i) subsistence skills, such as food gathering, hunting and food preparation;^22^ (ii) child feeding complementary to breastfeeding;^25^^,^^32^^,^^34^^,^^40^ (iii) school meals or nutrition policies;^26^^,^^29^ (iv) access to and choice of food;^35^ (v) food security;^17^^,^^23^^,^^37^^,^^38^ (vi) diet diversity or quality;^17^^,^^21^ (vii) adherence to a prescribed diet;^27^ (viii) calorie or food group consumption (e.g. fruit and vegetables, animal protein or processed food);^21^^,^^24^^,^^28^^,^^33^^,^^36^^,^^39^ and (ix) macro- and micro-nutrient intake.^30^^,^^32^ Physical activity outcomes included: (i) active travel (e.g. walking or cycling for transport);^19^ (ii) total physical activity; (iii) domains of physical activity (e.g. occupational or leisure);^20^^,^^26^^,^^28^ (iv) total sedentary behaviour; (v) domains of sedentary behaviour (e.g. television watching);^20^^,^^26^ and (vi) physical inactivity (e.g. not meeting physical activity guidelines).^16^^,^^18^^,^^21^^,^^33^ Several reviews also reported physical fitness.^20^^,^^28^

The reviews reported a range of hypothesized and demonstrated relationships between various factors and diet and physical activity. These were categorized using Dahlgren and Whitehead’s social model of health (Table 2). Little of the summarized evidence was related to distal factors in the category of international health, policy and politics in the social model of health and there were relatively few reported associations with physical activity in any category.^9^

**Table 2:** Factors associated with diet and physical activity in Africa and the Caribbean, scoping review of reviews, 1998–2019

Social model of health category^a^	Factors associated with diet	Factors associated with physical activity
**Distal factors**		
International health, policy and politics	Colonization;^17^ high-economic-value or cash crops;^17^^,^^24^ humanitarian aid (such as donated cereals);^17^ development aid and poverty reduction;^24^ gross domestic product;^24^ nutritional or epidemiological transition;^39^ dominance of major international retailers and producers;^23^ globalized (i.e. western) diet – high energy and low nutritional value;^23^ infectious diseases (including HIV/AIDS);^23^ dual burden of under- and overnutrition;^24^^,^^39^ and climate change or variability (e.g. erratic rainfall)^23^	Epidemiological transition^39^
General socioeconomic, cultural and environmental conditions	Access to, and availability of, food;^35^ price of food;^23^^,^^24^ individual purchasing power;^23^^,^^35^ availability of energy-rich, cheaper foods;^23^ frequency, quality or size of meals;^23^ socioeconomic status;^20^^,^^23^^,^^33^ parental socioeconomic status;^25^ mass media;^34^ cultural beliefs;^35^ extreme weather (e.g. drought);^17^^,^^19^ food security;^17^^,^^23^ wild food sources;^22^^,^^23^ indigenous vegetable crops;^23^ infectious disease;^23^ gendered roles;^21^^–^^24^ institutional exclusion of women (e.g. powerlessness, vulnerability and lack of control over assets);^23^ deagrarianization;^23^ urbanization;^17^^,^^39^ habitat loss;^23^ human–environment interactions;^23^ lack of desire to engage in agriculture (signal of poverty);^23^^,^^24^ and social grants (particularly for HIV/AIDS)^23^^,^^24^	Socioeconomic status;^33^ cultural heritage and gender disparity;^19^^,^^20^^,^^22^ weather (e.g. heavy rain disrupting travel across unbridged river);^19^ cultural practices and norms (e.g. running to school);^19^ urbanization;^39^ indoor leisure activities;^39^ technology (e.g. television, computer or mobile phone use);^39^ gendered roles;^19^ household responsibilities and work burden;^19^ punishment (including corporal) if late for school;^19^ fear of attack from people (e.g. violence, rape or harassment);^19^ dangerous vehicles;^19^ dangerous animals;^19^ and topography (such as rivers to cross or difficult terrain)^19^
**Proximal factors**		
Living and working conditions (including agriculture, food production, education, work environment, unemployment, water, sanitation, health-care services and housing)	Poverty;^23^^,^^24^ occupation;^33^ unemployment;^23^ distance from markets;^23^^,^^32^ market access for rural development;^23^ available land and land rights;^23^^,^^24^ geography (e.g. coastal versus inland, highlands versus lowlands, and particular regions or provinces);^17^ seasonality (particularly of fresh fruit and vegetables);^23^ locally grown produce;^23^^,^^24^^,^^32^ food quality;^32^ street-food nutritional composition;^36^ convenience and taste of food;^36^^,^^39^ fortified foods;^32^ urban versus rural areas;^23^^,^^24^ school meals;^24^^,^^29^^,^^34^^,^^35^ nutrition education interventions;^26^^,^^28^ cooking demonstrations;^25^^,^^40^ agricultural interventions (e.g. for poverty reduction);^24^ agricultural expertise and training;^23^^,^^24^ urban agriculture (including food gardens);^23^ home gardening;^25^ nutritional advice from health-care workers;^34^ road improvements;^24^ personal assets;^23^^,^^33^ education on nutrition and health;^33^ integration of nutrition education into existing curriculum;^26^^,^^28^ school physical environment;^26^ school nutrition policies (e.g. availability of healthy snacks);^26^^,^^28^ cognitive, behavioural or psychosocial approaches to nutrition;^26^^,^^27^^,^^40^ prompts or rewards for healthy food choices;^26^^,^^28^ parental education;^25^ water availability;^23^^,^^24^ agricultural inputs;^23^^,^^24^ household size and composition;^23^^,^^31^ household food allocation;^35^ food preparation techniques;^22^^,^^32^ antiretroviral medication (needs to be taken with food);^23^ antenatal and postnatal care;^25^ and multicomponent interventions^26^^,^^27^	Urban versus rural areas;^16^^,^^18^ built environment and perceived access to destinations (e.g. schools, shops and bus stops);^19^ lack of green space;^39^ unsafe neighbourhoods;^39^ multicomponent interventions;^26^^,^^27^ agricultural interventions (e.g. for poverty reduction);^24^ personal assets;^33^ education;^33^ integration of physical activity into existing curriculum;^26^^,^^28^ exercise classes or after-school sports;^26^ school travel time;^19^ school type (e.g. public versus private);^19^ school physical environment;^26^ physical activity equipment;^26^^,^^28^ and gendered roles^19^
Social and community networks	Social capital, networks, support and relationships;^23^ trust, reciprocity and exchange;^23^ exclusion and power imbalances;^23^ social meaning-making;^23^ church membership;^23^ collective action and cooperation (such as a savings club);^23^ self-esteem;^23^ social interaction and skills acquisition;^22^ perception of the consumption of healthy food;^39^ community-based platforms or committees;^24^^,^^32^ social behavioural change interventions;^35^ key influencers or family members;^35^ caregiver involvement;^26^ paternal involvement;^25^ maternal diet;^25^ knowledge of quantity of food to eat during pregnancy;^35^ advice from health-care professionals;^25^ intervention delivered by community members;^26^^,^^27^ peer support;^26^^–^^28^ counselling and communication skills;^35^ declining indigenous knowledge;^23^ strategies to procure food (e.g. selling assets);^23^ perspectives and experience of food security;^23^ consumer acceptability and perceptions of processed cereals;^23^ taboos, beliefs, rules and norms;^23^^,^^35^ and psychosocial determinants^39^	Cultural practices and norms (e.g. running to school);^19^ girls afraid of encounters with strangers;^19^ restriction of girls’ mobility after puberty;^19^ feeling travel to school is safe;^19^ insecure neighbourhoods;^19^ caregiver involvement;^28^ sport tournaments;^26^^,^^28^ peer training and support;^26^^–^^28^ perceived importance of physical activity;^39^ and psychosocial determinants^39^
Age, sex and constitutional factors	Sex;^21^^–^^23^^,^^31^^,^^34^ age;^22^^,^^32^ and infection status^24^	Sex;^16^^,^^18^^–^^20^ ethnicity;^16^ and age^18^^,^^19^

HIV/AIDS: human immunodeficiency virus and acquired immunodeficiency syndrome.

^a^ Factors reported in reviews as associated with diet or physical activity were categorized using Dahlgren and Whitehead’s social model of health.^9^

A wide range of associations were described, particularly for diet (Table 2). Several reviews reported that the shift to an urban, westernized lifestyle and diet and the threat of a competitive, globalized market were permeating influences.^23^^,^^24^^,^^29^ On diet, reviews that considered factors in the category of international health, policy and politics mentioned: the historic influence of colonization; humanitarian and development aid; the epidemiological transition; the transition to a western lifestyle and diet; the dual burdens of over- and undernutrition; infectious and chronic disease; and the impact of climate change. In addition, associations were described with: socioeconomic, cultural and environmental conditions, including access to food, the availability of food, prices, food security, deagrarianization and urbanization; living and working conditions, including education, poverty, household composition, land rights, skills, assets, rurality, and agricultural and school-based interventions; social and community networks, involving for example social capital, skills acquisition, peer support, key influencers, taboos and norms; and constitutional factors, particularly age and sex.

On physical activity, only one review described evidence on determinants in the most distal category of the social model of health (i.e. international health, policy and politics; Table 2). As expected, there were similarities and differences between the associations described for diet and physical activity. For example, both featured urbanization, socioeconomic status and gendered roles. In contrast, certain associations were described only for physical activity: (i) topography and climate; (ii) aspects of the built environment; (iii) dangerous traffic; (iv) fear of violent crime; (v) access to leisure facilities and green spaces; and (vi) restrictions on girls’ mobility after puberty.

Many reviews reported the heterogeneity and lack of standardization of the assessment methods used in the primary studies. For example, one review on food insecurity reported that the studies included used 26 distinct indicators of food insecurity and that many studies neither directly measured food insecurity nor adequately reported the measures they used.^23^ On physical activity, reviews typically reported that the primary studies tended to use self-report assessments and not objective assessments or measuring tools.^18^^–^^20^^,^^28^

## Discussion

We identified 25 reviews published between 1998 and 2019 that described factors associated with diet and physical activity in Africa and the Caribbean. Although our scoping review considered only evidence from these regions, our findings confirm that evidence is generally lacking from such settings on which to base policy and design interventions for improving diet and physical activity. Moreover, our findings are consistent with those of a previous study,^10^ which carried out a systematic review of research from low- and lower-middle-income countries published between 1990 and 2015 on the effect of interventions aligned with WHO’s “best buy” interventions on noncommunicable disease.^41^ They identified 36 studies, which covered only nine of the 83 low- and lower-middle-income countries. Only two of the 36, both from Pakistan, concerned diet and physical activity. In our study, we found no review from Africa or the Caribbean that summarized evidence relevant to WHO’s “best buy” interventions. Similarly, none of the literature we identified assessed primary research relevant to WHO’s global action plan targets on noncommunicable diseases or to targets set for the relevant sustainable development goals (SDGs).^4^^,^^42^

Although there may be research from Africa and the Caribbean that has not yet been reviewed, our findings suggest that, to date, policies on diet and physical activity are not informed by summarized research evidence on their determinants from these settings. This conclusion has two clear implications: (i) relevant primary research that has not yet been reviewed should be identified and evaluated; and (ii) new research should be undertaken to fill gaps in the evidence.

The policy responses and types of intervention required to improve health outcomes associated with diet and physical inactivity may be quite different in Africa and the Caribbean than in higher-income settings. In the absence of evidence indicating how different they need to be, current international guidance (e.g. WHO’s “best buy” interventions and recommendations in the global action plan on noncommunicable diseases) should be followed, so long as the interventions employed are robustly evaluated and can subsequently contribute to the evidence available from Africa and the Caribbean.^43^ Research funding bodies could help fill knowledge gaps and encourage the production of evidence summaries to guide policy. It would help if the terminology and definitions used for outcomes and their hypothesized determinants were much more consistent than we found in our study. In addition, international research networks that cover a range of different settings across Africa and the Caribbean could help develop and promote the high-quality, multidisciplinary research needed to address the complexity inherent in understanding how behavioural determinants vary between different contexts.^44^

In choosing to carry out a broad scoping review of factors associated with diet and physical activity and by adopting the review as the unit of analysis, our intention was to highlight gaps in the summarized literature (rather than in the primary literature) as an aid to policy-making. We did not directly look for primary research on the determinants of diet and physical activity, nor did we summarize policy documents. Consequently, our review does not indicate, for example, whether or not there exists a large number of primary research studies that have not yet been included in systematic appraisals of the evidence. Nor can we evaluate the degree to which existing policies are evidence-based; we can only comment on whether there is sufficient summarized evidence to inform those policies.

Our search strategy and the study’s conclusions were limited to factors that had a hypothesized or demonstrated association with behaviours affecting diet or physical activity. It is likely that, in some settings, academic research investigated factors associated with obesity or noncommunicable disease but did not explicitly categorize behaviour. Consequently, given that we were primarily interested in factors associated with behaviour rather than disease, our search strategy – though broad – could have missed some reviews of the determinants of diet and physical activity. Moreover, the cut-off date for inclusion in our review was 2019, which was just 4 years into the period covered by the SDGs. Most of the research included was, therefore, conducted during the era of the millennium development goals, which focused on undernutrition and did not stipulate any targets or indicators for noncommunicable disease.

Another limitation was that we did not appraise the quality of the reviews or the robustness of their evidence because our scoping review was intended primarily to map work in this area. Moreover, we identified papers only in English and may have missed reviews in other languages. We did not search the grey literature as our focus was on peer-reviewed academic journals. However, having identified gaps in the literature, we plan to include both academic and grey literature in a range of languages in future reviews.

In conclusion, our scoping review of reviews provides an overview of an important and rapidly evolving area of work. As our search strategy was kept broad by design, we found that a wide range of factors were reported to be associated with diet and physical activity in Africa and the Caribbean. However, evidence on which to base policy or to design interventions was lacking, which highlights the need for further reviews of the primary evidence to inform policy responses where the evidence exists, and to establish whether additional primary research is needed. As the modifiability of determinants of diet and physical activity and the feasibility of modifying them vary widely, future research should be aligned with policy targets and should evaluate the effectiveness of policy responses in different contexts.
